# Risk Factors for Middle East Respiratory Syndrome Coronavirus Infection among Healthcare Personnel

**DOI:** 10.3201/eid2211.160920

**Published:** 2016-11

**Authors:** Basem M. Alraddadi, Hanadi S. Al-Salmi, Kara Jacobs-Slifka, Rachel B. Slayton, Concepcion F. Estivariz, Andrew I. Geller, Hanan H. Al-Turkistani, Sanaa S. Al-Rehily, Haleema A. Alserehi, Ghassan Y. Wali, Abeer N. Alshukairi, Esam I. Azhar, Lia Haynes, David L. Swerdlow, John A. Jernigan, Tariq A. Madani

**Affiliations:** King Faisal Specialist Hospital and Research Center, Jeddah, Saudi Arabia (B.M. Alraddadi, H.S. Al-Salmi, H.H. Al-Turkistani, S.S. Al-Rehily, H.A. Alserehi, G.Y. Wali, A.N. Alshukairi);; Ministry of Health, Jeddah (B.M. Alraddadi, E.I. Azhar, T.A. Madani);; Centers for Disease Control and Prevention, Atlanta, Georgia, USA (K. Jacobs-Slifka, R.B. Slayton, C.F. Estivariz, A.I. Geller, L. Haynes, D.L. Swerdlow, J.A. Jernigan);; King Abdulaziz University, Jeddah (E.I. Azhar, T.A. Madani)

**Keywords:** Risk factors, MERS-CoV, Middle East respiratory syndrome coronavirus, healthcare personnel, healthcare workers, personal protective equipment, occupational health, viruses, zoonoses, respiratory infections

## Abstract

Healthcare settings can amplify transmission of Middle East respiratory syndrome coronavirus (MERS-CoV), but knowledge gaps about the epidemiology of transmission remain. We conducted a retrospective cohort study among healthcare personnel in hospital units that treated MERS-CoV patients. Participants were interviewed about exposures to MERS-CoV patients, use of personal protective equipment, and signs and symptoms of illness after exposure. Infection status was determined by the presence of antibodies against MERS-CoV. To assess risk factors, we compared infected and uninfected participants. Healthcare personnel caring for MERS-CoV patients were at high risk for infection, but infection most often resulted in a relatively mild illness that might be unrecognized. In the healthcare personnel cohort reported here, infections occurred exclusively among those who had close contact with MERS-CoV patients.

## Abstract 2

Infections occurred exclusively among personnel who had close contact with MERS-CoV patients.

Middle East respiratory syndrome coronavirus (MERS-CoV), first identified in 2012, has emerged as a cause of severe acute respiratory illness in humans. As of May 1, 2016, a total of 1,728 laboratory-confirmed cases, including 624 deaths, have been reported globally (*1*). All reported cases have been directly or indirectly linked to countries in or near the Arabian Peninsula, including a recent outbreak in South Korea resulting from a single imported case in a person with history of travel to the Middle East (*2*,*3*). Increasing evidence suggests that dromedary camels are a natural host for MERS-CoV and that camel-to-human transmission can occur, initiating short chains of human-to-human transmission (*4*–*7*). Numerous questions about the epidemiology of MERS-CoV remain unanswered.

Healthcare settings are important amplifiers of transmission (*6*,*8*,*9*). A 2014 case series of 255 MERS-CoV infections in Saudi Arabia found that 31% of cases occurred among healthcare personnel (HCP), and among case-patients who were not HCP, 87.5% had recent healthcare exposure (*9*). Current MERS-CoV infection control recommendations are based on experience with other viruses rather than on a complete understanding of the epidemiology of MERS-CoV transmission (*10*,*11*).

The World Health Organization recently issued an urgent call for studies to better understand risk factors for infection and transmission (*12*). Published case series of healthcare-associated MERS-CoV infections have major limitations, including lack of control groups and lack of serologic confirmation of infection status, leaving wide knowledge gaps, such as mode of and risk factors for transmission in healthcare settings, attack rate among HCP, and spectrum of illness for MERS-CoV infection (*13*). To address these gaps, we retrospectively studied MERS-CoV infection among a cohort of HCP in a hospital in Saudi Arabia.

## Methods

The study was conducted at King Faisal Specialist Hospital and Research Center (Jeddah, Saudi Arabia) during May–June 2014. This multispecialty hospital has 360 beds, including an 18-bed medical intensive care unit (MICU) and a 38-bed emergency department (ED). Seventeen patients with confirmed MERS-CoV infection were in the hospital during March 24–May 3, 2014. The hospital had no cases of MERS-CoV before March 24, 2014. All patients with suspected or confirmed MERS-CoV infection were placed in private rooms equipped with negative pressure ventilation. Patients in whom MERS-CoV infection was not suspected initially were transferred to negative-pressure rooms as soon as the diagnosis was suspected or confirmed. During the outbreak, all HCP who had contact with MERS-CoV cases were screened for symptoms and underwent testing for MERS-CoV RNA by real-time reverse transcription PCR (rRT-PCR) of nasopharyngeal swab specimens.

We assessed risk factors for a case, defined as a MERS-CoV antibody–positive serum sample from an HCP, among 3 cohorts of HCP. Two cohorts, 1 each from the ED and MICU, comprised all HCP who worked in those hospital units during March 24–May 14, 2014, the period during which those units treated patients known to have MERS-CoV infection. In addition, we included a cohort of all HCP who worked in a unit (neurology) that was not known to house any MERS-CoV patients during the study period.

Every healthcare worker in each cohort was recruited to enroll. Participants provided a serum sample and were interviewed by trained study personnel using a standardized questionnaire. Although HCP were from different cultural, language, and educational backgrounds, all spoke English fluently. All questionnaires were conducted in English. Interviews were conducted during May 28–July 10, 2014. In addition to age, sex, occupation, and co-morbidities, we collected information on signs, symptoms, and treatment from March 31, 2014, through the day of interview. Contacts with MERS-CoV patients were described, including patient care activities, duration of contact, and exposure to body fluids. Information about infection control training and use of personal protective equipment (PPE) during encounters with MERS-CoV patients was collected. We assessed exposures outside the hospital, including household exposures to persons with MERS-CoV, contact with animals, and travel.

Serum samples were screened for antibodies against MERS-CoV (Hu/Jordan-N3/2012) nucleocapsid (N) protein by ELISA. The recombinant MERS-CoV N indirect ELISA was developed by using a modified version of the HKU5.2 N ELISA previously described (*13*) Serum was considered positive when the optical density values were >0.36 (mean absorbance 405 nm of serum from US blood donors + 3 SD) with an assay specificity of 98.1% (544/555). Samples that were positive by ELISA were confirmed by immunofluorescence assay, microneutralization assay, or both (*14*). A positive serologic test result required confirmation by immunofluorescence assay or microneutralization assay. HCP whose serum sample tested positive for MERS-CoV antibodies were considered to have evidence of MERS-CoV infection (case-HCP); seronegative persons were considered uninfected.

We analyzed data using SAS version 9.3 (SAS Institute, Cary, NC, USA). As appropriate, we compared dichotomous variables using χ^2^ and Fisher exact tests. Cochran-Armitage tests for trend were used for ordinal variables. We performed multivariate logistic regression with backward stepwise elimination for exposures with univariate p<0.2. Variables with p<0.1 were retained in the final generalized linear model using a logit link to estimate risk.

We obtained written informed consent from all participants. The Institutional Review Board of the King Faisal Specialist Hospital and Research Centre approved the study.

## Results

Of 363 HCP eligible for the MICU (178 HCP), ED (137 HCP), and neurology unit (48 HCP) cohorts, 292 (80.4%) HCP were enrolled: 131 (73.5%) from the MICU, 127 (92.7%) from the ED, and 34 (70.8%) from the neurology unit. Of the 292 enrolled persons, 9 were excluded because serum specimens were unavailable.

For study participants who worked in units that treated MERS-CoV patients, the attack rate was 8.0% (20/250) and varied by hospital unit: MICU, 11.7% (15/128); ED, 4.1% (5/122). (The attack rate in the neurology unit, where no known MERS-CoV patients were treated, was 0% [0/33].) Attack rates in the MICU and ED also varied by occupation; radiology technicians had the highest attack rate (29.4% [5/17]), followed by nurses (9.4% [13/138]), respiratory therapists (3.2% [1/31]), and physicians (2.4% [1/41]). No clerical staff (7 participants) or patient transporters (14 participants) were seropositive. Most participants (64.4% [161/250]) were female; attack rate did not differ by sex (male 7.9%, female 8.1%; p = 0.95). The mean age of seropositive HCP was 40 years (range 29–59 years) and of seronegative HCP 37 years (range 18–66 years). 

The most common manifestations of illness among case-HCP were muscle pain, fever, headache, and dry cough (Table 1). These signs and symptoms, along with shortness of breath, occurred significantly more often among seropositive than among seronegative HCP. Seropositive HCP were also more likely to report gastrointestinal symptoms (p<0.001). Of the 20 case-HCP, 3 (15%) were asymptomatic, 12 (60%) had mild illness (symptomatic illness not requiring hospital admission), 2 (10%) had moderate illness (required hospital admission but not mechanical ventilation), and 3 (15%) had severe illness (required mechanical ventilation). All case-HCP survived, and all had been previously tested for MERS-CoV by rRT-PCR of nasopharyngeal swab specimens, but only 5 (25%) rRT-PCRs were positive.

**Table 1:** MERS-CoV symptoms reported by healthcare personnel, King Faisal Specialist Hospital and Research Center, Jeddah, Saudi Arabia, March–July 2014*

Symptom	Seropositive, no./No.† (%)	Seronegative, no./No.† (%)	p value
Muscle pain	13/20 (65.0)	66/260 (25.4)	0.0001
Fever	12/19 (63.2)	42/258 (16.3)	<0.0001
Dry cough	11/20 (55.0)	80/262 (30.5)	0.02
Headache	11/20 (55.0)	80/262 (30.5)	0.02
Diarrhea	7/20 (35.0)	21/262 (8.0)	0.0001
Nausea	7/20 (35.0)	18/262 (6.9)	<0.0001
Shortness of breath	7/20 (35.0)	32/261 (12.3)	0.005
Runny nose	6/19 (31.6)	92/263 (35.0)	0.76
Chills	6/20 (30.0)	23/261 (8.8)	0.003
Sore throat	5/20 (25.0)	118/263 (44.9)	0.08
Vomiting	4/20 (20.0)	10/262 (3.8)	0.01
Productive cough	3/18 (16.7)	39/263 (14.8)	0.74
Rash	1/20 (5.0)	4/259 (1.5)	0.26
None	3/20(15.0)	94/263 (35.7)	0.019

*MERS-CoV, Middle East respiratory syndrome coronavirus. †Denominator is the number of healthcare personnel who responded to the question.

Nineteen (95%) of 20 case-HCP reported having been in the same room as or within 2 meters of a patient known to be infected with MERS-CoV. The 1 seropositive HCP who had no MERS-CoV patient contact reported being in an automobile with a symptomatic person subsequently confirmed to have MERS-CoV infection. We therefore limited our analysis of risk factors, including PPE use, to any study participant who reported direct contact (i.e., within 2 meters) with MERS-CoV patients in the hospital (Table 2). Total time spent in a MERS-CoV patient’s room or handling the patient’s bedding, equipment, or fluids did not significantly differ between seropositive and seronegative HCP (p = 0.93), nor did the number of MERS-CoV patients cared for during the study period (median 3.0 and 5.0 patients for seropositive and seronegative HCP, respectively; p = 0.75). We found no association between animal contact and infection.

**Table 2:** RR of MERS-CoV seropositivity among persons with direct MERS-CoV patient contact, King Faisal Specialist Hospital and Research Center, Jeddah, Saudi Arabia, March–July 2014*

Condition/exposure	Condition/exposure present (AR, %)	Condition/exposure absent (AR, %)	RR (95% CI)	p value
Co-morbidity	8/68 (11.8)	11/156 (7.1)	1.67 (0.70–3.96)	0.30
Asthma	2/16 (12.5)	17/208 (8.2)	1.53 (0.39–6.04)	0.63
Allergic rhinitis	5/31 (16.1)	14/192 (7.3)	2.21 (0.86–5.71)	0.15
Chronic lung disease	0/2 (0)	19/222 (8.6)	–	1
Chronic heart disease	0/10 (0)	19/214 (8.9)	–	1
Diabetes mellitus	3/20 (15.0)	16/202 (7.9)	1.89 (0.60–5.95)	0.39
Chronic kidney disease	0	19/224 (8.5)	–	–
End-stage renal disease requiring hemodialysis	0	19/224 (8.5)	–	
Immunosuppressive condition	0/2 (0)	19/222 (8.6)	–	1
Autoimmune disease	0	19/224 (8.5)	–	
Pregnant	0/3 (0)	13/137 (9.5)	–	1
Exposure to a MERS-CoV patient	18/208 (8.7)	1/16 (6.3)	1.38 (0.20–9.72)	1
Taking vital signs	10/122 (8.2)	9/102 (8.8)	0.92 (0.39–2.20)	1
Providing medication	10/115 (8.7)	9/109 (8.3)	1.05 (0.44–2.49)	0.91
Placing urinary catheter	3/49 (6.1)	16/175 (9.1)	0.67 (0.20–2.21)	0.77
Bathing	7/76 (9.2)	12/148 (8.1)	1.14 (0.47–2.77)	0.78
Feeding	6/70 (8.6)	13/154 (8.4)	1.02 (0.40–2.56)	0.97
Lifting, positioning	14/131 (10.7)	5/93 (5.4)	1.99 (0.74–5.33)	0.16
Emptying bedpan	8/71 (11.3)	11/153 (7.2)	1.57 (0.66–3.73)	0.31
Changing linen	11/109 (10.1)	8/115 (7.0)	1.45 (0.61–3.47)	0.40
Providing injection	10/94 (10.6)	9/130 (6.9)	1.54 (0.65–3.63)	0.32
Placing intravascular device	10/73 (13.7)	9/151 (6.0)	2.30 (0.98–5.41)	0.05
Performing hemodialysis	2/37 (5.4)	17/187 (9.0)	0.59 (0.14–2.46)	0.75
Taking medical history	6/98 (6.1)	13/126 (10.3)	0.59 (0.23–1.50)	0.26
Performing physical exam	9/140 (6.4)	10/84 (11.9)	0.54 (0.23–1.27)	0.15
Participating in surgery	0/6 (0)	19/218 (8.7)	–	1
Drawing blood	11/119 (9.2)	8/105 (7.6)	1.21 (0.51–2.90)	0.66
Collecting respiratory laboratory specimens	9/111 (8.1)	10/113 (8.9)	0.92 (0.39–2.17)	0.84
Performing radiograph	8/60 (13.3)	11/164 (6.7)	1.99 (0.84–4.70)	0.12
Processing clinical specimen	3/22 (13.6)	16/202 (7.9)	1.72 (0.54–5.45)	0.41
Visiting in the hospital	5/70 (7.1)	14/154 (9.1)	0.79 (0.29–2.10)	0.63
Present in room when any of the following procedures were performed on a MERS-CoV patient	16/177 (9.0)	3/47 (6.4)	1.42 (0.43–4.66)	0.77
Manipulation of oxygen face mask or tubing†	13/157 (8.3)	6/67 (9.0)	0.92 (0.37–2.33)	0.87
Airway suction†	9/128 (7.0)	10/96 (10.4)	0.67 (0.29–1.60)	0.37
Noninvasive ventilation†	9/105 (8.6)	10/119 (8.4)	1.02 (0.43–2.41)	0.96
Manual ventilation†	5/90 (5.6)	14/134 (10.5)	0.53 (0.20–1.42)	0.23
Nebulizer treatments†	9/103 (8.7)	10/121 (8.3)	1.05 (0.45–2.50)	0.90
Intubation†	7/105 (6.7)	12/119 (10.1)	0.66 (0.27–1.63)	0.36
Cardiopulmonary resuscitation†	6/87 (6.9)	13/137 (9.5)	0.73 (0.29–1.84)	0.50
High-frequency oscillatory ventilation†	1/19 (5.3)	18/205 (8.8)	0.60 (0.08–4.25)	1
Chest tube insertion or removal	0/20 (0)	19/204 (9.3)	–	0.23
Insertion of nasogastric tubes	5/64 (7.8)	14/160 (8.8)	0.89 (0.34–2.38)	1
Insertion of peripheral line	9/110 (8.2)	10/114 (8.8)	0.93 (0.39–2.21)	0.87
Insertion of central venous line	4/67 (6.0)	15/157 (9.6)	0.62 (0.22–1.81)	0.44
Chest physiotherapy	3/49 (6.1)	16/175 (9.1)	0.67 (0.20–2.21)	0.77
Tracheostomy care	5/55 (9.1)	14/169 (8.3)	1.10 (0.41–2.91)	0.79
Bronchoscopy†	0/4 (0)	19/220 (8.6)	–	1
Extubation†	1/4 (25.0)	18/220 (8.2)	3.06 (0.53–17.67)	0.30
Any aerosol-generating procedure	15/172 (8.7)	4/52 (7.7)	1.13 (0.39–3.27)	1
Direct contact with any blood, body fluid, or excretion of MERS-CoV patient	5/78 (6.4)	14/145 (9.7)	0.66 (0.25–1.77)	0.46
Blood	4/53 (7.6)	15/171 (8.8)	0.86 (0.30–2.48)	1
Sputum	4/52 (7.7)	15/172 (8.7)	0.88 (0.31–2.54)	1
Urine	3/27 (11.1)	16/197 (8.1)	1.37 (0.43–4.39)	0.71
Feces	3/32 (9.4)	16/192 (8.3)	1.12 (0.35–3.64)	0.74
Other fluids	1/8 (12.5)	18/216 (8.3)	1.50 (0.23–9.89)	0.51
Smoking	10/85 (11.8)	9/139 (6.5)	1.82 (0.77–4.29)	0.17
Currently smokes tobacco	4/52 (7.7)	15/172 (8.7)	0.88 (0.31–2.54)	1
Smoked tobacco in the past	7/40 (17.5)	8/141 (5.7)	3.08 (1.12–7.99)	0.02
Other exposures				
Respiratory pathogen infection control training	15/202 (7.4)	4/17 (23.5)	0.32 (0.12–0.85)	0.05
MERS-CoV infection control training	7/138 (5.1)	12/82 (14.6)	0.35 (0.14–0.85)	0.01
Same room or <2 m of any other hospitalized patient with pneumonia or respiratory illness	15/188 (8.0)	2/29 (6.9)	1.16 (0.28–4.80)	1
Contact with persons other than patients sick with fever or respiratory illness	5/76 (6.6)	14/145 (9.7)	0.68 (0.26–1.82)	0.61
Contact with persons other than patients who were MERS-CoV positive	2/36 (5.6)	16/177 (9.0)	0.61 (0.15–2.56)	0.74
Hijab wear while working (women)	2/11 (18.2)	11/132 (8.3)	2.18 (0.55–8.64)	0.26
Any travel	9/87 (10.3)	10/132 (7.6)	1.37 (0.58–3.22)	0.48

*AR, attack rate; MERS-CoV, Middle East respiratory syndrome coronavirus; RR, relative risk. †Aerosol-generating procedure.

We assessed HCP’s self-reported use of PPE during care of MERS-CoV patients, stratified by type of equipment and type of patient interaction (Table 3). HCP who reported always covering their nose and mouth with either a medical mask or N95 respirator had lower risk for infection than did HCP reporting not always or never doing so, although this association was statistically significant only among HCP present in the room where aerosol-generating procedures were conducted. HCP who reported always using a medical mask for direct patient contact were ≈3 times more likely to have MERS-CoV infection than were HCP who reported not always or never using a medical mask (98% of whom reported always or sometimes using an N95 respirator), a trend that was not statistically significant (p = 0.10). Conversely, those who reported always using N95 respirators for direct patient contact were less likely to be seropositive, a trend that approached statistical significance (p = 0.07).

**Table 3:** PPE used by healthcare personnel during care of MERS-CoV patients, King Faisal Specialist Hospital and Research Center, Jeddah, Saudi Arabia, March–July 2014*

PPE used, contact type	Always wore PPE,† no. seropositive/total‡ (%)	Sometimes/never wore PPE,§ no. seropositive/total‡ (%)	RR (95% CI)	p value
Gloves	18/197 (9.1)	0/21 (0)	NA	NA
Gown	11/139 (7.9)	7/79 (8.9)	0.89 (0.36–2.21)	0.81
Eye protection				
Direct contact	1/47 (2.1)	17/165 (10.3)	0.21 (0.03–1.51)	0.13
Aerosol-generating procedure	3/62 (4.8)	11/100 (11.0)	0.44 (0.13–1.51)	0.25
Covering of nose and mouth with medical mask or N95 respirator¶				
Direct patient contact	11/151 (7.3)	7/66 (10.6)	0.69 (0.28–1.69)	0.43
Aerosol-generating procedures	8/133 (6.0)	6/32 (18.8)	0.32 (0.12–0.86)	0.03
Medical mask				
Direct patient contact	9/69 (13.0)	9/142# (6.3)	2.06 (0.86–4.95)	0.10
Aerosol-generating procedures	5/81 (6.2)	8/76 (10.5)	0.59 (0.20–1.71)	0.39
N95 respirator				
Direct patient contact	6/116 (5.2)	12/101** (11.9)	0.44 (0.17–1.12)	0.07
Aerosol-generating procedures	5/90 (5.6)	9/73 (12.3)	0.45 (0.16–1.29)	0.16

*MERS-CoV, Middle East respiratory syndrome coronavirus; NA, not applicable; PPE, personal protective equipment; RR, relative risk. †Reported always wearing PPE indicated in table row when caring for MERS-CoV patients. ‡Total number of healthcare personnel who responded to the question about PPE. §Reported not always or never wearing PPE indicated in table row when caring for MERS-CoV patients. ¶Reported use of medical mask and N95 respirator were not mutually exclusive categories; therefore the number of healthcare personnel reporting always wearing either an N95 respirator or always wearing a medical mask does not sum to the “covering of nose and mouth with medical mask or N95 respirator” category. #Of the 142 who reported not always or never wearing a medical mask, 139 (98%) reported always or not always wearing an N95 respirator (55% always, 45% not always). **Of the 101 who sometimes or never wore an N95 respirator, 96 (95%) reported always or not always wearing a medical mask (35% always, 65% not always).

Because medical mask and N95 respirator use were strongly and inversely correlated, we built separate multivariate models, one that assessed risk for medical mask use (model 1) and another that assessed risk for N95 respirator use (model 2). In both models, having participated in infection control training that included information about MERS-CoV prevention was associated with a significant and strong protective effect, and there was a strong but statistically insignificant trend toward increased risk among smokers. In model 1, HCP who reported always using a medical mask for direct MERS-CoV patient care were significantly more likely to be seropositive than those who reported not always or never wearing a medical mask (almost all of whom sometimes or always wore an N95 respirator) (relative risk [RR] 2.73, 95% CI 0.99–7.54). This model also included past or current smoking (RR 2.54, 95% CI 0.93–6.96) and participation in MERS-CoV infection control training (RR 0.28, 95% CI 0.10–0.80). In model 2, N95 respirator use was associated with a strong protective trend; HCP who always used an N95 respirator for direct MERS-CoV patient care were 56% less likely to be seropositive than were those who reported not always or never using an N95 respirator (almost all of whom sometimes or always wore a medical mask) (RR 0.44, 95% CI 0.15–1.24). This model also included past or current smoking (RR 2.51, 95% CI 0.92–6.87) and participation in MERS-CoV infection control training (RR 0.33, 95% CI 0.12–0.90).

## Discussion

We report this seroepidemiologic study to quantify the risk for MERS-CoV infection among HCP. The findings have important implications for infection control practice. Our results suggest that the attack rate of MERS-CoV infection among healthcare workers is substantially higher than that in previous reports that used nonserologic methods of detection (*15*–*17*). The spectrum of illness appears to be broader than previously described; infection caused a relatively mild illness in most cases. Infections occurred almost exclusively among HCP having close contact with a MERS-CoV patient.

Most HCP in this cohort reported always covering their nose and mouth with a medical mask or N95 respirator when caring for a MERS-CoV patient, which appeared to protect against infection among HCP participating in aerosol-generating procedures. When we stratified by type of mask, we observed an increased risk for MERS-CoV infection among HCP who reported always using medical masks and, conversely, a lower risk among those who reported always using N95 respirators. Taken together, these results raise the hypothesis that short-range aerosol transmission might have factored in transmission. Previous studies suggest that some respiratory viruses (e.g., influenza, severe acute respiratory syndrome coronavirus, rhinovirus) that are transmitted primarily by droplets and/or contact might simultaneously be spread through aerosol under certain conditions and perhaps by certain patients (*18*–*22*). Aerosol transmission in close proximity to the patient might not necessarily be accompanied by long-range transmission because the risk for such transmission might be affected by the infectious dose, the amount of aerosolized particles generated at the source, and the rate of biologic decay of the agent (*22*). We found no evidence of long-range aerosol transmission. Until additional information about the mode of MERS-CoV transmission is available, it seems prudent to take precautions against aerosol spread in healthcare settings when feasible to do so.

The combined attack rate for HCP who worked in units known to house patients with MERS-CoV infection (8%) was substantially higher than that in previous studies, which described attack rates for HCP of <1% (*15*–*17*). These prior studies did not use serologic methods to detect infection but rather relied on rRT-PCR of nasopharyngeal swabs. All 20 seropositive HCP in our study were screened with nasopharyngeal swabs, and only 5 (25%) of these tests showed evidence of MERS-CoV by rRT-PCR. Therefore, screening for viral shedding using nasopharyngeal swabs might be an insensitive method for detecting infection, perhaps because of variability in timing of samples in relationship to exposure, and studies relying solely on this method of case detection might underestimate attack rates.

Our study suggests that almost all MERS-CoV infection among HCP occurs among those having close contact with patients known to be infected with MERS-CoV. We observed the highest attack rates among radiology technicians, followed by nurses. We hypothesize that radiology technicians most likely were exposed while obtaining portable chest radiographs, a procedure that requires close contact (e.g., positioning the patient for cassette placement) with patients who might be likely to have worsening respiratory status and be highly contagious. We identified no seropositive HCP who worked in the unit not known to house any MERS-CoV patients, suggesting that the background rate of MERS-CoV infection among HCP was low in the absence of known exposure to infected patients and that the virus was not circulating widely among staff.

HCP who had undergone infection control training specific to MERS-CoV had a lower risk for infection. This finding underscores the critical need for adequate infection control training, especially in settings with ongoing transmission of epidemiologically important pathogens.

We observed a broad spectrum of illness among HCP, and in most cases illness was relatively mild. Most illnesses were characterized by myalgia, fever, headache, and dry cough. Gastrointestinal symptoms were present in 50% of infected HCP; and 3 (15%) reported no symptoms. Most seropositive HCP with symptoms sought care, but only a small minority were recognized as having MERS-CoV infection. All 20 infected HCP survived, and only 5 required hospitalization. The spectrum of illness we observed was broader than that described in previous case series of MERS-CoV infection (*15*,*23*,*24*), which probably were biased toward identifying patients with more severe illness because testing for MERS-CoV infection has largely been triggered by case definitions requiring evidence of pneumonia (*10*). The observation that most MERS-CoV infections among HCP are likely to be relatively mild and unrecognized has potentially important implications for infection control practice. Although little is known about risk for transmission from persons with mild MERS-CoV-infection, HCP with unrecognized MERS-CoV infection might be a reservoir for transmission to hospitalized patients who are more susceptible to severe illness because of underlying illnesses. Transmission from persons with unrecognized MERS-CoV infections might have contributed to the major role healthcare-associated transmission has played in the epidemiology of MERS-CoV (*6*,*8*,*9*). Thus, control of transmission in healthcare settings might depend on maintaining a low threshold for suspicion of MERS-CoV infection among exposed HCP and other persons with a relatively mild viral syndrome.

Our study did not identify strong associations with underlying chronic illnesses, most likely because the prevalence of such conditions was low (<10%) in this population. HCPs with a history of smoking had a risk for infection almost 3 times that of nonsmokers. We found no association between MERS-CoV infection and sex. Most case series to date have demonstrated a male predominance among case-patients (*15*,*23*,*24*), but our study suggests this association might be explained by social and behavioral factors that increase exposure to MERS-CoV, rather than a sex-specific difference in biological susceptibility.

Our study has several strengths. We compared MERS-CoV infected and uninfected HCP to determine risk factors for acquiring infection during patient care. The use of serologic testing to determine infection status enabled unbiased case ascertainment, an examination of the full spectrum of disease, and a comparison of the risks associated with a wide range of specific patient care activities.

Our study also has limitations. First, questionnaires were administered several weeks after possible exposures, and therefore the potential exists for recall bias. Recall bias can limit assessment of important variables, such as frequency of exposure and duration of contact during specific procedure. However, HCP and interviewers were unaware of their serologic status at the time of interview; their answers would not have been influenced by knowledge of these results. Moreover, symptoms of illness were unlikely to have introduced systematic bias to responses because most uninfected and infected groups reported illness. Second, we used only 1 serum sample for serologic testing. Because of the retrospective nature of our study, baseline serologic tests were not conducted, and therefore the potential exists for false-positive results. However, seroprevalence of MERS-CoV antibodies in Saudi Arabia is low (0.15%), making misclassification bias unlikely (*25*). Third, infected asymptomatic HCP could serve as a potential source of infection to other HCP. Given the retrospective nature of our study, we were not able to characterize these potential exposures. Fourth, as is common with early studies of emerging infectious diseases, sufficiently powering studies can be difficult. Whether negative findings were true null findings or due to small sample sizes is unclear.

In conclusion, we report results of a seroepidemiologic study to quantify risk for MERS-CoV infection among HCP. The attack rate appears to be substantially higher than that in prior reports that used nonserologic methods of detection. Infection in this population most often results in mild illness that might be overlooked; programs to identify and exclude ill HCP who have been exposed to patients with MERS-CoV might help eliminate this reservoir for transmission. Our findings also suggest N95 respirators might be more protective against MERS-CoV infection while in close contact with an infected patient and highlight the possible role of short-range aerosol transmission of MERS-CoV in healthcare settings. Education about standard and MERS-CoV infection control practices appears to be protective, suggesting that adherence to basic practices can effectively prevent MERS-CoV infection among HCP.
